# The Paediatric Glaucoma Diagnostic Ability of Optical Coherence Tomography: A Comparison of Macular Segmentation and Peripapillary Retinal Nerve Fibre Layer Thickness

**DOI:** 10.3390/biology10040260

**Published:** 2021-03-25

**Authors:** Mael Lever, Christian Halfwassen, Jan Darius Unterlauft, Nikolaos E. Bechrakis, Anke Manthey, Michael R. R. Böhm

**Affiliations:** 1 Department of Ophthalmology, University Hospital Essen, 45122 Essen, Germany; christian.halfwassen@uk-essen.de (C.H.); Nikolaos.bechrakis@uk-essen.de (N.E.B.); anke.manthey@uk-essen.de (A.M.); michael.boehm@uni-due.de (M.R.R.B.); 2 Department of Ophthalmology, University Hospital Leipzig, 04103 Leipzig, Germany; JanDarius.Unterlauft@medizin.uni-leipzig.de

**Keywords:** childhood glaucoma, macular segmentation, retinal nerve fibre layer, optical coherence tomography

## Abstract

**Simple Summary:**

Optical coherence tomography (OCT) is an established ophthalmologic diagnostic tool to visualise vital retinal structures. In glaucoma, it is used to quantify the thickness decrease in the peripapillary retinal nerve fibre layer (pRNFL) and in the macula. While glaucoma management in adults incorporates traditional clinical parameters as well as instrumental methods such as OCT, guidelines for paediatric glaucoma focus on conventional methods. Even though some reports encouraging a broader use of OCT in children are present, its diagnostic potential in this particular population has still not been sufficiently analysed. To address this, the present study compares the glaucoma discriminative ability of OCT measurements of the pRNFL and macular layers in a paediatric population. The results indicate a reduction of the pRNFL and of inner macular layer thickness in glaucoma eyes, as well as a high correlation with the presence of glaucoma. The glaucoma discriminative ability can be maximised combining either all pRNFL sectors or the thickness results of the three innermost macular layers, even though sensitivity remains moderate. In conclusion, the OCT measurements of the pRNFL and macular thickness have a strong ability to diagnose paediatric glaucoma. However, OCT should be used in addition to conventional diagnostic tools rather than as a standalone method.

**Abstract:**

Paediatric glaucoma leads to a decreased thickness of the peripapillary retinal nerve fibre layer (pRNFL) and of the macula. These changes can be precisely quantified using spectral domain-optical coherence tomography (SD-OCT). Despite abundant reports in adults, studies on the diagnostic capacity of macular SD-OCT in paediatric glaucoma are rare. The aim of this study was to compare the glaucoma discriminative ability of pRNFL and macular segment thickness in paediatric glaucoma patients and healthy children. Data of 72 children aged 5–17 years (glaucoma: 19 (26.4%), healthy: 53 (73.6%)) examined with SD-OCT (SPECTRALIS^®^, Heidelberg Engineering) were analysed retrospectively. The thickness of pRNFL sectors and of macular segment subfields were compared between diseased and healthy participants. Areas under the receiver-operating characteristic curves (AUC), sensitivity, and specificity from logistic regression were used to evaluate the glaucoma discriminative capacity of single and combined pRNFL and macular segments’ thickness. The results revealed a reduced thickness of the pRNFL and of the three inner macular layers in glaucoma patients, which correlates highly with the presence of glaucoma. The highest glaucoma discriminative ability was observed for the combination of pRNFL sectors or inner macular segments (AUC: 0.83 and 0.85, respectively), although sensitivity remained moderate (both 63% at 95% specificity). In conclusion, while confirmation from investigations in larger cohorts is required, SD-OCT-derived pRNFL and macular thickness measurements seem highly valuable for the diagnosis of paediatric glaucoma.

## 1. Introduction

Paediatric glaucoma is characterised by a damage of retinal ganglion cells (RGC), the optic nerve, and of other ocular structures due to elevated intraocular pressure [1]. Although it is rare, paediatric glaucoma may be the cause for estimated 5% of blindness in children. The prevalence depends on the region and on its aetiology [2]. Today, paediatric glaucoma is diagnosed in the presence of elevated intraocular pressure (IOP), characteristic morphologic changes to the optic nerve head (ONH), corneal abnormalities (e.g., Haab striae, enlarged diameter), progressive myopia or axial length, and/or glaucomatous visual field (VF) defects [3]. Most of these parameters need to be assessed by an experienced clinician as they depend on the patient’s cooperation [4] and can be mistaken for physiological normal variants (e.g., megalocornea, megalopapilla). 

Optical coherence tomography (OCT) is considered a promising additional diagnostic method in paediatric glaucoma. It can be used to mitigate the subjectivity and reproducibility concerns of traditional clinical diagnostic parameters. In particular, OCT provides a contactless, highly reproducible [5,6], objective, and 3-dimensional visualisation of the retina and of the optic nerve head (ONH) [7]. OCT uses interferometry of low-coherence light from a diode or laser reflected from the retinal layers compared to a reference mirror. With spectral-domain OCT (SD-OCT), the increased axial resolution and computer assisted segmentation help deliver detailed information about ONH and macular morphology [8]. In adult glaucoma, OCT measurements of the peripapillary retinal nerve fibre layer (pRNFL) thickness is an established tool for diagnosis and follow-up [9] as the glaucoma-induced loss of RGC leads to a thinning of the neuroretinal rim around the ONH [10]. In children with glaucoma, a correlation between the thinning of the pRNFL and pathologic ONH cupping was previously shown [8,11]. Moreover, reports of a correlation between pRNFL thickness and the presence of glaucoma advocate for the potential diagnostic value of OCT for paediatric glaucoma [12,13].

In addition to this, several aspects justify the inclusion of morphologic macular parameters in glaucoma management. The macula contains about 50% of the RGC population of the eye [14], and the thickness of the selected macular layers correlate with pRNFL thickness [15,16]. The structural simplicity of the macula, its lack of blood vessels, and small interindividual variability allow for a more precise and reliable examination compared to the ONH [17]. Recent clinical studies described alterations of the macular architecture (in particular of its three innermost layers) in adult glaucoma patients [18,19]. Consequently, a comparable glaucoma discriminating ability was found for macular segmentation measurements as for pRNFL thickness [20,21]. In paediatric glaucoma, a reduction of the macular volume [8,12] and inner layer thickness [22,23] has been documented, but the diagnostic ability of these measurements has rarely been investigated [24].

The purpose of this study was thus to evaluate the diagnostic ability of pRNFL and macular segment thickness measurements in a group of childhood glaucoma patients and healthy children.

## 2. Materials and Methods

### 2.1. Study Design

Retrospective chart analysis of patients aged < 18 years referred to the Department of Ophthalmology of the University Hospital Essen, Germany, between 2016 and 2018 due to childhood glaucoma or glaucoma suspicious optic nerve head (ONH) morphology (e.g., high ONH excavation, megalopapilla). Patients were included after an OCT of the macula and of the pRNFL was acquired on the same day. Exclusion criteria were the presence of any systemic diseases (in particular cardiovascular or neurologic), preterm birth (birth before 37 completed weeks of gestation), and any non-glaucomatous condition apart from strabismus (especially optic atrophy, papilledema, amblyopia). Charts lacking data about IOP, visual acuity (VA), anterior segment examination, and/or fundoscopy, e.g., due to a lack of participation, were excluded from the study. Data of the right eye were selected for further analysis whenever possible. This study was conducted in accordance with the 1964 Declaration of Helsinki and was approved by the ethics committee of the University Hospital Essen, Germany (approval number: 16-7114-BO, on 24 August 2017).

### 2.2. Glaucoma Diagnosis and Related Parameters

The presence of childhood glaucoma was evaluated in accordance to the recommendations of the ninth Consensus Report of the World Glaucoma Association [25] and the recommendation of the Childhood glaucoma Research Network [3]. Diagnosis was confirmed in the presence of at least two of the following criteria: elevated IOP measurements, ONH morphology (cupping, focal rim loss), glaucoma typical corneal changes (Haab striae), enlarged corneal diameter, increasing axial length or myopia. If necessary, examinations were performed under general anaesthesia.

On the day of OCT examination, a comprehensive ophthalmic examination was performed, including a review of past medical history and current therapy, determination of visual acuity with correction according to cycloplegic objective refraction, slit-lamp examination of the anterior segment (if applicable), fundoscopy (including evaluation of ONH linear cup-to-disc ratio, CDR) and stereoscopic ONH photography, measurement of IOP (Goldmann applanation tonometer, Haag-Streit, Bern, Switzerland), central corneal thickness (CCT; Canon TX-20P tonometer), and a visual field examination using 30-2 static automated perimetry (SAP) (Twinfield 2, OCULUS Optikgeräte, Wetzlar, Germany). Together, results were interpreted independently by two consultant ophthalmologists of our department’s glaucoma division.

### 2.3. OCT Measurements

**Figure 1:**
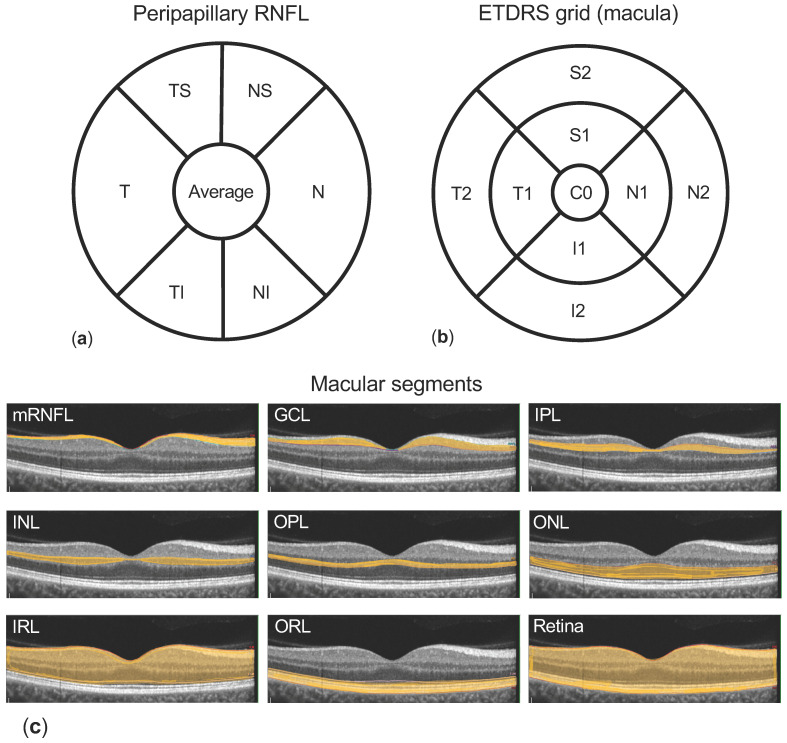
Methodology. (**a**) shows the peripapillary retinal nerve fibre layer (pRNFL) quadrants and sectors (T: temporal, N: nasal, superior separated into TS: temporal superior and NS: nasal superior, and inferior separated into TI: temporal inferior and NI nasal inferior) measured by the optical coherence tomography (OCT) software. Macular segments (**c**) are separated semi-automatically (macular retinal nerve fibre layer (mRNFL), ganglion cell layer (GCL), inner plexiform layer (IPL), inner nuclear layer (INL), outer plexiform layer (OPL), outer nuclear layer (ONL)–grouped as inner retinal layers (IRL), retinal pigment epithelium (RPE), and outer retinal layers (ORL)), and the thickness of each layer is reported using the Early Treatment Diabetic Retinopathy Study (ETDRS) grid 1, 3.5, 6 mm (**b**) containing nine subfields (C0: centre, S1 and S2 superior, N1 and N2 nasal, I1 and I2 inferior, and T1 and T2 temporal).

Measurements of the macula and pRNFL were obtained by spectral domain-optical coherence tomography (SD-OCT) using a SPECTRALIS^®^ SD-OCT (Heidelberg Engineering, Heidelberg, Germany). Corneal curvature values (c-curve) were known for all patients. At least two consecutive examinations of sufficient image quality (quality score ≥ 20) were obtained; thickness results were extracted and averaged. For pRNFL measurements, a circular scan of 3.5 mm containing 768 A-scans across 360° is centred to the ONH. The manufacturer’s software (version 1.10.2.0, Heidelberg Engineering, Heidelberg, Germany) divides the disc into four symmetrical 90° quadrants (superior S, nasal N, inferior I, and temporal T) and averages them (total average thickness). Additionally, the superior and inferior quadrants are divided in 45° sectors (temporal superior TS, nasal superior NS, nasal inferior NI, and temporal inferior TI, respectively) (Figure 1a). The later introduced anatomic positioning system was not used in this study.

For OCT examination of the macula, 25 single horizontal axial scans centred to the fovea are acquired. Using the manufacturers’ software, semi-automated image segmentation is calculated to obtain individual retinal layer thicknesses: total retinal thickness (“retina”), macular retinal nerve fibre layer (mRNFL), ganglion cell layer (GCL), inner plexiform layer (IPL), inner nuclear layer (INL), outer plexiform layer (OPL), outer nuclear layer (ONL), and retinal pigment epithelium (RPE) (Figure 1c). Additionally, the retinal segments mRNFL, GCL, IPL, INL, OPL, and ONL are combined as inner retinal layers (IRL). The photoreceptor layer and RPE are combined as outer retinal layers (ORL). Results of the semi-automated segmentation were inspected and corrected manually when required. Thickness results were divided into nine subfields using the 1, 3.5, 6 mm Early Treatment Diabetic Retinopathy Study (ETDRS) grid (Figure 1b). Thickness values of each subfield were exported using a software plug-in provided by the device manufacturer.

### 2.4. Statistical Analysis

The numerical data were collected in Microsoft Excel (Microsoft, Redmond, WA, USA). Normality was examined using the D’Agostino and Pearson normality tests. Mean values were compared applying Student’s *t*-test, or the Mann–Whitney U test, if appropriate. Correlation between parameters were evaluated calculating Pearson or Spearman correlation factors, when appropriate. Simple and multivariate logistic regression analyses were performed to evaluate the correlation between macular layer thickness and the presence of glaucoma; goodness-of-fit was interpreted from the 95% confidence interval of the coefficients (95% CI) and its odds ratio (OR), Tjur’s pseudo R^2^, and the *p*-value of the Likelihood ratio test. Univariate and multivariate logistic regression were calculated to estimate the correlation between the thickness of pRNFL sectors and of the macular segments; goodness-of-fit was evaluated using the 95% CI of the coefficients, R^2^, and the *p*-value of the F-test. Statistical analyses were performed using Prism 8.3 (GraphPad, La Jolla, CA, USA), except for the power calculation, for which we used G*Power software version 3.1 for a two-sided *t*-test, and α = 0.05 [26]. In the results section of this article, numeric results are presented as “mean ± standard deviation” or as “median (confidence level of the median (CL))”, when appropriate. In general, statistical significance was assumed for *p* < 0.05.

## 3. Results

### 3.1. Patients’ Characteristics

A total of 72 patients aged 5.9 to 17.9 (11.9 ± 3.6 years) were included in this study. A total of 26 patients (36.1%) were ≤10 years old. Female gender was slightly underrepresented (44.4%, *n* = 32). Among the 19 (26.4%) paediatric glaucoma patients in this study, 42.1% (*n* = 8) were aged ≤10 years (Table 1). A post hoc analysis of statistical power returned high values for the comparison of both pRNFL and macular thickness (e.g., pRNFL average thickness 1-β = 0.98; inner superior subfield of the ganglion cell layer (S1 of GCL) 1-β = 0.98). Juvenile open angle glaucoma (JOAG) was the main cause of glaucoma (*n* = 7, 36.8%), followed by primary congenital glaucoma (PCG) (*n* = 5, 26.3%), and glaucoma associated to acquired conditions (GAC, *n* = 6, 31.6%), including glaucoma following cataract surgery (GFC) (*n* = 4, 21.1%). The detailed distribution of glaucoma aetiologies is displayed in Table 2. The 53 (73.6%) healthy patients constitute the control group. The median BCVA was 0.0 LogMAR (CL: 96.0%). The overall mean IOP was 15.7 ± 4.8 (glaucoma: 18.7 ± 7.2 mm Hg; control: 14.5 ± 2.8 mm Hg, *p* = 0.0007). Antiglaucomatous therapy in the glaucoma group consisted of a median of 2 (CL: 98.1%) topical and/or systemic agents. The mean ONH diameter was 1.65 ± 0.24 mm (glaucoma: 1.55 ± 0.20 mm; control: 1.68 ± 0.25, *p* = 0.051). The median linear CDR of the whole cohort was 0.6 (CL: 96%) (glaucoma: 0.8 (CL: 98%); control: 0.6 (CL 97%), *p* = 0.030). Additional epidemiologic data are presented in Table 1.

The pRNFL thickness was significantly thinner in glaucoma patients compared to healthy controls in all quadrants and sectors (e.g., total average thickness: glaucoma: 82.8 ± 19.8; control 98.7 ± 6.93, *p* < 0.0001) except in the nasal quadrant (glaucoma: 68.0 ± 18.3; control: 75.0 ± 12.5, *p* = 0.072) (Table 3).

### 3.2. Comparison of Macular Layer Thickness in Glaucoma Patients and Healthy Individuals

After segmentation of retinal layers within the macula, the thickness of single ETDRS subfield in all segments was compared between glaucoma patients and healthy controls. In general, a reduced layer thickness is observed in glaucoma patients. However, this was not entirely consistent across all layers and subfields and important differences were found. GCL thickness is reduced in glaucoma patients compared to healthy children in all subfields except C0 (e.g., inferior I2 glaucoma: 28.6 ± 5.9; control: 33.2 ± 4.2 µm, *p* = 0.0005). IPL thickness is lower in glaucoma patients compared to controls in the superior, inferior, and temporal subfields (e.g., inferior I2 glaucoma: 24.2 ± 4.3; control: 26.4 ± 3.4 µm, *p* = 0.024) but not in the nasal subfield and C0. Furthermore, measurements of the entire retina and of the inner retinal layers (IRL) share significant thickness differences between glaucoma and healthy subjects: in the superior, inferior, and outer temporal subfields (e.g., retina: inferior I2 glaucoma: 277.6 ± 21.6; control: 293.7 ± 18.2 µm, *p* = 0.0024) but not in the nasal, T1, and C0 subfields. Regarding inner layers, the mRNFL, thickness differences appear only in S2 (glaucoma: 32.3 ± 10.4; control: 37.0 ± 4.3 µm, *p* = 0.0082) and I2 (glaucoma: 34.1 ± 11.6; control: 39.7 ± 6.0 µm, *p* = 0.011). Contrarily, in the inner and outer nuclear layers (INL and ONL, respectively), no difference of thickness can be observed. Finally, OPL thickness is significantly increased in the inferior, outer nasal, and temporal subfields in glaucoma patients (e.g., I2 glaucoma: 29.6 ± 5.7; control: 26.4 ± 3.0 µm, *p* = 0.0026). Selected data are presented in Table 4 and all results are available in Table S1.

### 3.3. Glaucoma Discriminative Ability of pRNFL and Macular Thickness Measurements

**Figure 2:**
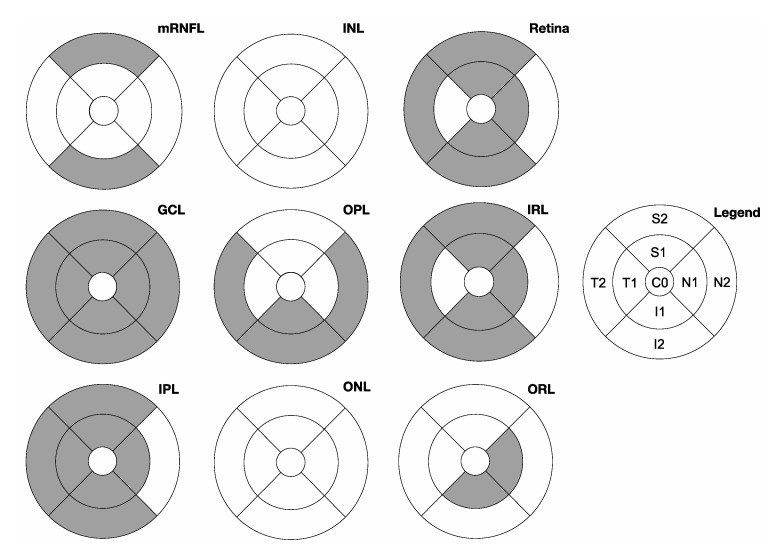
Overview of the correlation between the thickness of subfields of all macular layers and the presence of glaucoma. The ETDRS subfields with a significant correlation with glaucoma diagnosis by univariate logistic regression are filled in grey. Abbreviations: mRNFL: macular retinal nerve fibre layer; GCL: ganglion cell layer; IPL: inner plexiform layer; INL: inner nuclear layer; OPL: outer plexiform layer; ONL: outer nuclear layer–grouped as inner retinal layers (IRL); ORL: outer retinal layers.

To evaluate the correlation between both the pRNFL and the macular layers’ thickness with the diagnosis of paediatric glaucoma, logistic regression analyses were performed. In univariate logistic regression, each pRNFL sector and single macular subfields were used as independent variable. For multivariate logistic regression, all pRNFL sectors or macular subfields were used simultaneously. The most notable results are presented in Table 5; Figure 2 provides a graphical overview of the correlation for each macular segment and subfield. All numerical results can be found in Supplementary Table S2.

Performing univariate logistic regression with the pRNFL thickness reveals a high correlation with the presence of glaucoma. This correlation is the highest in the inferior quadrant and temporal inferior sector (TI) with R^2^ = 0.33 (both) and *p* = 0.005 (inferior quadrant). The combination of the pRNFL thickness of all sectors using multivariate logistic regression shows an even higher correlation (R^2^ = 0.39, *p* ≤ 0.0001).

Univariate logistic regression also reveals a correlation between macular segment thickness and the presence of glaucoma for all ETDRS subfields except C0 in GCL (e.g., outer inferior subfield I2: R^2^ = 0.19, *p* = 0.0005). In IPL, a correlation is also visible with all ETDRS subfields except for C0 and N2 (e.g., outer inferior subfield I2: R^2^ = 0.08, *p* = 0.023). For the mRNFL thickness, univariate logistic regression reveals a correlation only for S2 and I2 (e.g., outer inferior subfield I2: R^2^ = 0.14, *p* = 0.003). In the OPL, the increased thickness observed in glaucoma patients leads to a correlation with the diagnosis in the N2, I1, I2, and T2 subfields (e.g., outer inferior subfield I2: R^2^ = 0.12, *p* = 0.0039). In both retinal nuclear layers (INL, ONL), no correlation between single subfields and glaucoma diagnosis can be stated. Concerning the entire retinal thickness and IRL, a correlation appears in all subfields except for C0, N2, and T1 (e.g., retina: outer inferior subfield I2: R^2^ = 0.14, *p* = 0.0017).

**Figure 3:**
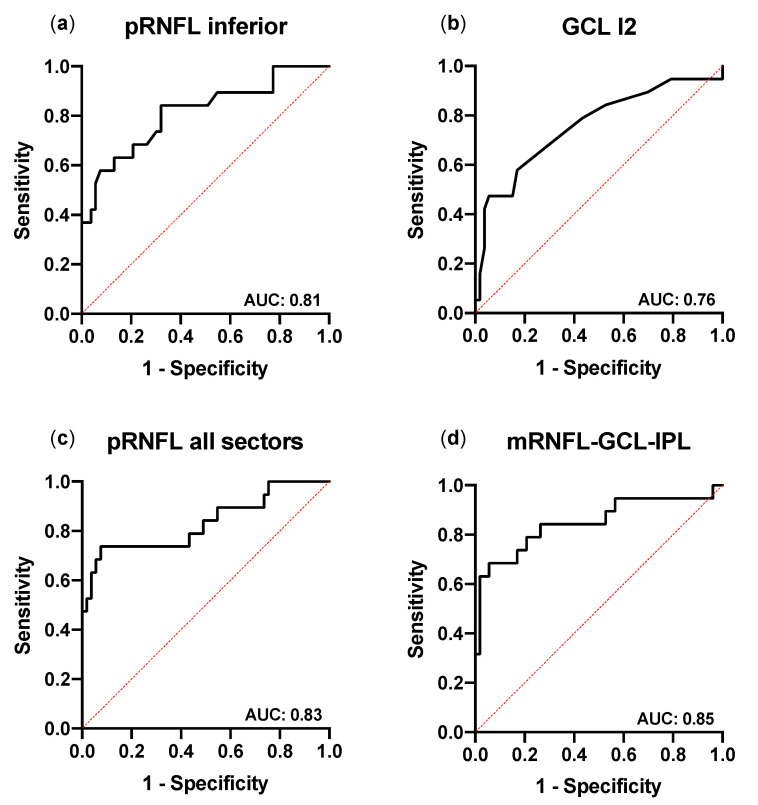
Receiver-operating characteristic curves of the best performing pRNFL and macular parameters for the discrimination between glaucoma and healthy. The receiver-operating characteristic (ROC) curves in the univariate logistic regression for glaucoma identification of the best performing pRNFL (inferior quadrant) and macular (outer-inferior (I2) subfield of the ganglion cell layer (GCL)) areas are shown in (**a**,**b**), respectively. The ROC curves of the multivariate logistic regression of all pRNFL sectors or combining the mean thickness of the inferior and superior subfields of the mRNFL (macular retinal nerve fibre layer), GCL, and inner plexiform layer (IPL) are shown in (**c**,**d**), respectively. The AUC (area under the ROC curve) values are presented in the lower-right corner of each graph.

To evaluate the potential of pRNFL and macular segment thickness measurements to discriminate between glaucoma and healthy eyes, receiver-operating characteristic (ROC) curves were examined (Figure 3a) and their respective area under the ROC curve (AUC) was calculated (Table 5). For pRNFL thickness, AUC is lowest in the nasal quadrant (AUC: 0.63, 95% CI: 0.47–0.79) and highest in the inferior quadrant and temporal inferior sector (TI) (both AUC: 0.81, 95% CI: 0.69–0.93). The AUC of the multivariate logistic regression combining all pRNFL quadrants is higher than the AUC for single pRNFL sectors (AUC: 0.83, 95% CI: 0.71–0.96; Figure 3c).

The analysis of the glaucoma discriminative ability of macular layers reveals a highest value in GCL for the outer inferior subfield I2 (AUC: 0.76, 95% CI: 0.62–0.89; Figure 3b), followed by I1, S1, T2, and N2. The AUC for subfields of IPL is lower than in GCL. In detail, the highest correlation of IPL is found in I2 and T2 (both AUC: 0.68, 95% CI: 0.53–0.83). The results of the entire retinal thickness are comparable with highest AUC found in I2 (I2: AUC: 0.71, 95% CI: 0.56–0.85), followed by I1 and T2. In addition, multivariate logistic regression analysis combining the mean thickness of the inferior (I1 and I2) and superior (S1 and S2) subfields of the mRNFL, GCL, and IPL returns an AUC substantially higher (0.85, 95% CI: 0.73–0.97; Figure 3d) than the AUC of single ETDRS subfields or single pRNFL sectors. This AUC is comparably high to the AUC of combined pRNFL quadrant thickness.

Finally, the comparison of the sensitivity of univariate and multivariate logistic regression shows that, at fixed specificity, the sensitivity is moderate for single pRNFL sectors (e.g., inferior sector: 42% sensitivity at 95% specificity) and for selected subfields of the GCL (e.g., outer inferior subfield I2: 42% sensitivity at 95% specificity). In other macular layers, the sensitivity is lower. When combining pRNFL sectors, the sensitivity of multivariate regression is higher but still moderate (sensitivity: 63%, at 95% specificity). Similarly, the combination of the superior and inferior subfields of the mRNFL, GCL, and IPL returns a sensitivity of up to 63% (at a specificity of 95%) (Table 6).

## 4. Discussion

This study addresses the role of pRNFL and retinal layer thickness for identifying paediatric glaucoma by presenting a comparison of SD-OCT measurements of the pRNFL and of macular segmentation in childhood glaucoma patients and healthy children. The main findings of this work are:

1. The thickness of both pRNFL and selected macular segments is reduced in glaucoma patients;
2. The reduced thickness of the pRNFL and of macular segments correlate positively with the presence of glaucoma;
3. The thickness of the pRNFL and of macular segments shows a high discriminative ability in paediatric glaucoma.

Identifying childhood glaucoma is a challenging task. Generally, the diagnosis is confirmed based on clinically assessed parameters such as IOP, ONH morphology, changes to the cornea, and, if possible, functional (e.g., perimetric) tests [25]. The availability and reliability of these parameters often depend on the patient’s cooperation [27]. Reliable SD-OCT measurements also require the patient’s compliance and can therefore be difficult to obtain from young children. However, OCT, as an objective, fast, and contact free method, has been shown to provide precise and reliable measurements throughout visits [6,8]. These characteristics are particularly advantageous for the management of paediatric glaucoma. In the present study, the observation of SD-OCT measurements of 72 children (19 with paediatric glaucoma, 59 healthy controls) as young as 6 years of age, reveals a significantly reduced pRNFL thickness in glaucoma patients compared to the healthy controls. This is in line with previous results on adults [28] and children [8,29].

In addition to examining the pRNFL, OCT is also used to visualise macular morphology. The technology is used in a variety of ophthalmic and neurologic conditions. With the introduction of time-domain OCT technology, a reduction of the total macular volume and thickness in glaucoma patients was observed [8,12]. The development of SD-OCT enhanced the capacity to analyse macular architecture, particularly through the measurement of single retinal layers and segments [30]. In the presented cohort, the entire thickness of the macula was significantly reduced in children with glaucoma compared to healthy age-matched controls. In detail, the analysis of macular segments principally reveals a reduced thickness in the layers GCL, IPL, and mRNFL. In contrast, the retinal nuclear layers INL and ONL show no thickness difference between diseased and healthy subjects. This has already been described in previous studies, stating that morphologic glaucomatous damage to the macula affected primarily its three inner most layers (mRNFL, GCL, and IPL) [22,23]. This can be explained by the glaucoma-induced loss of retinal ganglion cell axons, cell bodies, and dendrites, which constitute the mRNFL, GCL and IPL, respectively [15]. Silverstein et al. [22] reported an increase in INL thickness in paediatric glaucoma patients compared to healthy children. In our study, a significantly increased thickness of the OPL, which is adjacent to the INL, is witnessed in glaucoma patients. Finally, the presented lack of difference in ONL thickness between diseased and healthy eyes correlates with missing histological alterations in ONL and photoreceptor layer in glaucoma affected adults [31]. This aspect has been controversially discussed as other studies reported histological alterations of photoreceptors in the context of glaucoma [32,33]. However, this discrepancy between SD-OCT and histological analysis could be explained by a decreasing image quality of SD-OCT in deeper retinal layers and by the missing alterations in the mild to moderate glaucoma stages in the present cohort. 

The presented results argue for a broader use of macular examination in the management of paediatric glaucoma. However, this study has several limitations. First, the number of glaucoma patients included is relatively small, in general and compared to the number of controls included in the study. Even though the measured statistical power of our analyses is good, this prevented the creation of more complex multivariate regression models and the differentiated comparison of results by glaucoma aetiology. In addition, average disease severity evaluated by perimetry was low and the relative distribution of glaucoma aetiologies in our cohort differs from their reported global incidences; here, JOAG is overrepresented at the expense of PCG [1]. This is explained by a selection bias caused by the difficulty to include young PCG patients due to frequent media opacity and reduced fixation, both negatively affecting OCT image quality. This bias makes a generalisation of the presented results to the diversity of paediatric glaucomas hazardous.

The glaucoma identifying capacity of pRNFL thickness measurements is well documented in adults [34,35] and has also been reported in children [12,13]. The present results corroborate the previous findings where a strong correlation was found between pRNFL thickness and the presence of glaucoma. Consequent analyses of the AUC of logistic regression and its sensitivity and specificity results suggest a high ability of pRNFL thickness to discriminate between glaucoma and healthy eyes. As previous reports [12], this diagnostic potential is highest for the inferior quadrant and inferotemporal sector.

Further, the thickness differences of macular segments between childhood glaucoma patients and healthy children were analysed to evaluate their potential to discriminate between healthy and diseased eyes. The presented logistic regression results reveal a strong correlation between segment thickness and the presence of glaucoma. Appearing mostly in the inner macular layers, in particular in the GCL and IPL where the AUC is high, this observation is in line with a previous analysis on PCG patients [24]. Investigations on adult glaucoma patients also showed similar results with highest disease identifying ability found for the three innermost macular layers (mRNFL, GCL, and IPL), often summarised as the ganglion cell complex (GCC) [19,20]. In the present study, the combination of the inferior and superior subfields of these three layers provide a higher AUC than the univariate analyses. However, a difference between children and adult patients is that in adults the highest AUC was reported for the mRNFL [18], whereas in our cohort and the study by Morales-Fernandez [24], the mRNFL had a lower AUC than the GCL and IPL. This could be explained by a possible reduced reliability of segmentation and thickness measurements of the mRNFL, GCL, and IPL in children due to their thinness. However, this hypothesis needs further investigation. In addition, the analysis of single macular layer thickness in our cohort reveals a correlation with the presence of glaucoma in deeper layers such as the OPL or the inner retinal layers. While these results are new, they lack consistency; hence, their clinical relevance is unclear.

Recent studies compared the glaucoma discriminative ability of pRNFL and macular thickness measurements. A higher sensitivity of pRNFL thickness measurements was reported compared to the GCC analysis in adult glaucoma [15,21]. Other studies propose a diagnostic advantage when including OCT analyses of selected macular parameters, particularly in advanced stages; in severe glaucoma, a floor effect of pRNFL thickness can be observed, which is absent in the macula [36]. Additionally, macular OCT is considered more reliable in high myopia, where myopic morphological alterations affect the ONH more heavily than the macula, thus impacting the reliability of pRNFL measurements more so than the macular [37]. In a previous study with PCG patients, AUC was slightly higher for pRNFL measurements (e.g., supero-temporal sector) compared to the best glaucoma discriminative macular parameter (S2 of the GCL) [24]. In the present study, the results are in line with the discussed studies, showing higher AUC for the best performing pRNFL sectors (inferior quadrant and TI) than the best performing macular subfield (I2 of GCL). This supports the hypothesis that pRNFL thickness has a small diagnostic advantage over the analysis of single macular layers thickness in children. In our study however, the sensitivity of these best performing papillary and macular parameters is very similar at various specificity levels. The potential advantage of pRNFL measurements can be further relativised when examining multivariate logistic regression. There, the AUC and sensitivity of the combination of mRNFL, GCL, and IPL subfield thickness is on par with the combination of all pRNFL quadrants. Together, the high AUC of peripapillary and macular OCT measurements reported here indicate a high capacity of these methods to correctly identify glaucomatous eyes, particularly in a screening situation. The sensitivity levels of up to 63% (at a specificity of 95%) in the present cohort, or 0.65–0.75 in a previous study [15], can only be considered moderate. This is insufficient to allow the use of OCT as a standalone diagnostic method for suspected paediatric glaucoma. Still, these results represent an important continuation of previous observations about the importance of macular segmentation for diagnosing paediatric glaucoma.

## 5. Conclusions

The present investigation reveals a combined thickness reduction of the pRNFL and macula in paediatric glaucoma. Using SD-OCT, single pRNFL and macular segmentation parameters showed a high glaucoma discriminative ability in our paediatric population. However, additional analyses suggest that the combination of all pRNFL quadrants or of inner macular layers can further increase the diagnostic potential of OCT. These results require further investigation in larger, prospective cohorts but advocate for a broader implementation of pRNFL measurements and macular segmentation in paediatric glaucoma management. The creation of a normative pRNFL and macular thickness database including paediatric subjects could enhance the reliability of these methods and therefore should be of high priority.

## Figures and Tables

**Table 1:** Epidemiologic and general ophthalmologic characteristics of patients.

Parameter		Value	*p*-Value ^1^
Patients	(*n*)	72	
Gender	male:female% (*n*)	55.6:44.4% (40:32)	
Diagnosis	glaucoma:healthy% (*n*)	26.4:73.6% (19:53)	
Eye	right:left% (*n*)	87.5:12.5% (63:9)	
Age	mean ± SD (*y*)	11.9 ± 3.6	
Glaucoma		11.2 ± 3.5	0.33
Healthy		12.2 ± 3.5	
Range	(*y*)	5.5–17.9	
≤10 years	*%* (*n*)	36.1% (26)	
>10 years	*%* (*n*)	63.9% (46)	
BCVA	median (CL) (LogMar)	0.0 (96.0%)	
IOP	mean ± SD (mm Hg)	15.7 ± 4.8	
Glaucoma		18.7 ± 7.2	**0.0007**
Healthy		14.5 ± 2.7	
ONH horizontal diameter	mean ± SD (mm)	1.65 ± 0.24	
Glaucoma		1.55 ± 0.20	0.051
Healthy		1.68 ± 0.25	
Linear CDR	median (CL)	0.6 (96%)	
Glaucoma		0.8 (98%)	**0.030**
Healthy		0.6 (97%)	
Perimetry (MD)	mean ± SD (dB)	2.7 ± 4.1	
Glaucoma		4.3 ± 5.5	0.131
Healthy		2.4 ± 3.7	
Follow-up time	mean ± SD (*m*)	20.0 ± 14.2	

^1^*p*-values of *t*-test or Mann–Whitney U test (when appropriate), significant when *p* < 0.05 (bold, underlined). Follow-up time represents the time since glaucoma diagnosis or the first visit (for cases or controls, respectively). Abbreviations: BCVA: best corrected visual acuity; IOP: intraocular pressure; ONH: optic nerve head; CDR: cup-to-disc ratio (by fundoscopy and/or ONH photography); y: years; mm Hg: millimetre of mercury; MD: mean deviation; dB: decibel; SD: standard deviation; CL: confidence level.

**Table 2:** Distribution of glaucoma aetiologies.

Diagnosis/Aetiology	*n*
Glaucoma patients	19
Primary congenital glaucoma (PCG)	5 (26%)
Juvenile primary open angle glaucoma (JOAG)	7 (37%)
Glaucoma associated with non-acquired ocular anomalies (GNAO)	1 (5%)
Glaucoma associated with non-acquired systemic anomalies	0 (0%)
Glaucoma associated with acquired conditions (GAC)	6 (32%)
Glaucoma after cataract (GFC)	4 (21%)
GAC others	2 (11%)

The table shows the relative distribution of glaucoma aetiologies in our cohort of 19 paediatric glaucoma patients. The classification was made using recommendations of the Childhood Glaucoma Research Network.

**Table 3:** Peripapillary retinal nerve fibre layer (pRNFL) thickness is reduced in glaucoma patients.

pRNFL Sector Thickness	Glaucoma		Healthy		*p*-Value ^1^
	Mean ± SD	95% CI	Mean ± SD	95% CI	
Average thickness	82.8 ± 19.8	73.2–92.3	98.7 ± 6.93	96.8–100.6	**<0.0001**
Superior	98.3 ± 35.1	81.4–115.2	122 ± 12.9	118.2–125.3	**<0.0001**
Temporal superior (TS)	108 ± 38.6	89.1–126.3	138 ± 15.4	133.6–142.1	**<0.0001**
Nasal superior (NS)	88.9 ± 34.3	72.4–105.4	106 ± 18.5	100.6–110.8	**0.0097**
Nasal	68.0 ± 18.3	59.2–76.4	75.0 ± 12.5	71.5–78.4	0.0717
Inferior	99.4 ± 28.0	86.0–112.9	128 ± 15.1	123.8–132.1	**<0.0001**
Nasal inferior (NI)	87.6 ± 26.9	74.7–100.6	110 ± 22.5	104.2–116.6	**0.0006**
Temporal inferior (TI)	111 ± 34.9	94.4–128.1	146 ± 17.3	140.8–150.4	**<0.0001**
Temporal	63.8 ± 15.6	56.3–71.4	70.8 ± 10.7	67.9–73.8	**0.0342**

^1^*p*-values of *t*-test or Mann–Whitney U test (when appropriate) significant when *p* < 0.05 (bold, underlined). Abbreviations: SD: standard deviation; CI: confidence interval; pRNFL: peripapillary retinal nerve fibre layer.

**Table 4:** Thickness of selected macular layers is reduced in glaucoma patients compared to healthy controls.

Macular Segment Thickness	Glaucoma	Healthy	*p*-Value ^1^
	Mean ± SD	Mean ± SD	
Ganglion cell layer (GCL)			
Central (C0)	20.8 ± 8.6	19.4 ± 7.9	0.62
Inner superior (S1)	44.1 ± 11.3	52.2 ± 6.2	**0.0002**
Outer superior (S2)	29.6 ± 5.8	33.9 ± 3.5	**0.0002**
Inner nasal (N1)	45.1 ± 12.9	52.9 ± 5.9	**0.0006**
Outer nasal (N2)	31.2 ± 7.8	36.4 ± 4.7	**0.001**
Inner inferior (I1)	42.8 ± 11.8	51.2 ± 7.0	**0.0004**
Outer inferior (I2)	28.6 ± 5.9	33.2 ± 4.2	**0.0005**
Inner temporal (T1)	40.5 ± 12.4	46.8 ± 6.0	**0.0047**
Outer temporal (T2)	29.3 ± 7.9	36.5 ± 4.6	**<0.0001**
Inner plexiform layer (IPL)			
Central (C0)	23.4 ± 7.1	23.2 ± 5.3	0.93
Inner superior (S1)	37.0 ± 6.7	40.7 ± 4.4	**0.0089**
Outer superior (S2)	25.2 ± 4.1	27.4 ± 3.0	**0.015**
Inner nasal (N1)	40.3 ± 6.1	42.3 ± 2.9	0.061
Outer nasal (N2)	26.1 ± 4.7	27.9 ± 3.3	0.079
Inner inferior (I1)	35.5 ± 8.4	40.1 ± 4.7	**0.0046**
Outer inferior (I2)	24.2 ± 4.3	26.4 ± 3.4	**0.024**
Inner temporal (T1)	36.9 ± 8.7	40.1 ± 4.1	**0.040**
Outer temporal (T2)	28.3 ± 5.7	31.7 ± 3.1	**0.0019**
Retina			
Central (C0)	287.1 ± 31.5	281.3 ± 28.9	0.47
Inner superior (S1)	331.2 ± 25.9	343.3 ± 14.7	**0.015**
Outer superior (S2)	289.0 ± 21.1	301.4 ± 15.2	**0.0078**
Inner nasal (N1)	338.5 ± 20.1	346.5 ± 13.9	0.065
Outer nasal (N2)	305.6 ± 29.9	316.5 ± 17.1	0.060
Inner inferior (I1)	323.3 ± 26.8	339.5 ± 21.5	**0.010**
Outer inferior (I2)	277.6 ± 21.6	293.7 ± 18.2	**0.0024**
Inner temporal (T1)	318.4 ± 25.2	327.5 ± 13.8	0.057
Outer temporal (T2)	274.9 ± 18.0	288.2 ± 16.3	**0.0041**

^1^*p*-values of *t*-test or Mann–Whitney U test (when appropriate) significant when *p* < 0.05 (bold, underlined). Abbreviations: SD: standard deviation.

**Table 5:** The thickness of the pRNFL and of selected macular layer subfields correlate with the presence of glaucoma.

Sector/Subfield	Coefficient	95% CI	OR	R^2^	*p*-Value ^1^	AUC	95% CI
Peripapillary retinal nerve fibre layer (pRNFL)							
Multivariate logistic regression							
All sectors				0.39	**<0.0001**	0.83	0.71–0.96
Univariate logistic regression							
Superior	−0.12	−0.20–−0.057	0.89	0.30	**0.0011**	0.75	0.60–0.90
Temporal superior (TS)	−0.046	−0.080–−0.020	0.96	0.21	**0.0021**	0.67	0.51–0.83
Nasal superior (NS)	−0.047	−0.080–−0.023	0.95	0.26	**0.0010**	0.72	0.57–0.88
Nasal	−0.029	−0.055–−0.0066	0.97	0.11	**0.0169**	0.61	0.44–0.78
Inferior	−0.035	−0.076–0.0025	0.97	0.054	0.077	0.63	0.47–0.79
Nasal inferior (NI)	−0.076	−0.13–−0.039	0.93	0.33	**0.0005**	0.81	0.69–0.93
Temporal superior (TI)	−0.042	−0.071–−0.017	0.96	0.18	**0.0023**	0.73	0.59–0.88
Temporal	−0.067	−0.11–−0.034	0.94	0.33	**0.0007**	0.81	0.69–0.93
Superior	−0.053	−0.11–−0.0054	0.95	0.079	**0.0429**	0.64	0.48–0.80
mRNFL-GCL-IPL							
multivariate logistic regression							
Superior and inferior subfields (S1, S2, I1, I2)				0.42	**<0.0001**	0.85	0.73–0.97
Macular retinal nerve fibre layer (mRNFL)							
Univariate logistic regression							
Inner superior (S1)	−0.027	−0.14–0.016	0.97	0.010	0.31	0.53	0.36–0.70
Outer superior (S2)	−0.11	−0.20–−0.028	0.90	0.13	**0.0048**	0.64	0.47–0.82
Inner inferior (I1)	−0.028	−0.14–0.011	0.91	0.013	0.25	0.54	0.37–0.70
Outer inferior (I2)	−0.090	−017–−0.027	0.91	0.14	**0.0030**	0.68	0.52–0.84
Ganglion cell layer (GCL)							
Univariate logistic regression							
Inner superior (S1)	−0.11	−0.20–−0.045	0.89	0.18	**0.0006**	0.74	0.60–0.89
Outer superior (S2)	−0.22	−0.37–−0.091	0.8	0.19	**0.0004**	0.71	0.57–0.86
Inner inferior (I1)	−0.098	−0.17–−0.039	0.91	0.17	**0.0009**	0.75	0.60–0.89
Outer inferior (I2)	−0.2	−0.35–−0.084	0.81	0.19	**0.0005**	0.76	0.62–0.89
Inner plexiform layer (IPL)							
Univariate logistic regression							
Inner superior (S1)	−0.12	−0.25–−0.026	0.88	0.096	**0.0126**	0.66	0.51–0.80
Outer superior (S2)	−0.19	−0.37–−0.033	0.83	0.086	**0.0173**	0.67	0.52–0.83
Inner inferior (I1)	−0.11	−0.22–−0.030	0.89	0.11	**0.0072**	0.67	0.50–0.84
Outer inferior (I2)	−0.17	−0.34–−0.022	0.84	0.08	**0.0233**	0.68	0.53–0.83

^1^*p*-values for the coefficient of the log-likelihood ratio test of logistic regression analysis, statistically significant when *p* < 0.05 (bold, underlined). Abbreviations: 95% CI: 95% confidence interval; OR: odds’ ratio; R^2^: Tjur’s pseudo R^2^ factor; AUC: area under the receiver operating characteristic curve.

**Table 6:** Sensitivity and specificity of selected pRNFL and macular areas.

Sector/Subfield	Sensitivity at		
	Specificity ≥ 80%	Specificity ≥ 90%	Specificity ≥ 95%
Peripapillary retinal nerve fibre layer (pRNFL)			
Multivariate logistic regression			
All sectors	74%	74%	63%
Univariate logistic regression			
Average	63%	53%	42%
Superior	53%	42%	42%
Inferior	68%	58%	42%
mRNFL–GCL–IPL			
Multivariate logistic regression			
Superior and inferior subfields (S1, S2, I1, I2)	74%	68%	63%
Macular retinal nerve fibre layer (mRNFL)			
univariate logistic regression			
Inner superior (S1)	42%	21%	11%
Outer superior (S2)	53%	47%	42%
Inner inferior (I1)	32%	21%	11%
Outer inferior (I2)	47%	37%	26%
Ganglion cell layer (GCL)			
univariate logistic regression			
Inner superior (S1)	63%	32%	32%
Outer superior (S2)	47%	42%	42%
Inner inferior (I1)	63%	42%	26%
Outer inferior (I2)	58%	47%	42%
Inner plexiform layer (IPL)			
univariate logistic regression			
Inner superior (S1)	32%	26%	26%
Outer superior (S2)	58%	37%	16%
Inner inferior (I1)	42%	37%	32%
Outer inferior (I2)	47%	37%	16%

## Data Availability

The data supporting the reported results can be requested from the corresponding author on reasonable request.

## Supplementary Materials

The following are available online at https://www.mdpi.com/2079-7737/10/4/260/s1, Table S1: Comparison of the thickness of macular segments and subfields of paediatric glaucoma patients and healthy controls; Table S2: Univariate and multivariate logistic regression analysis between the pRNFL or macular segment thickness and the presence of paediatric glaucoma.
